# Type 1 diabetes mellitus and SARS‐CoV‐2 in pediatric and adult patients – Data from the DPV network

**DOI:** 10.1111/1753-0407.13332

**Published:** 2022-11-28

**Authors:** Bastian Raphael Büttner, Sascha René Tittel, Clemens Kamrath, Beate Karges, Katharina Köstner, Andreas Melmer, Elke Müller‐Roßberg, Friederike Richter, Tilman R. Rohrer, Reinhard W. Holl

**Affiliations:** ^1^ Department of Pediatrics Saarland University Medical Center Homburg Saar Germany; ^2^ Institute for Epidemiology and Medical Biometry, Zentralinstitut für Biomedizinische Technik (ZIBMT) Ulm University Ulm Germany; ^3^ German Center for Diabetes Research (DZD) Munich‐Neuherberg Germany; ^4^ Center of Child and Adolescent Medicine Justus Liebig University Giessen Germany; ^5^ Division of Endocrinology and Diabetes, Medical Faculty RWTH Aachen University Aachen Germany; ^6^ German Center for Pediatric and Adolescent Rheumatology Garmisch‐Partenkirchen Germany; ^7^ Department of Diabetes, Endocrinology, Clinical Nutrition and Metabolism, Inselspital, Bern University Hospital University of Bern Bern Switzerland; ^8^ Department of Pediatric Endocrinology and Diabetes Children's Hospital Esslingen Germany; ^9^ Department of Pediatrics Garmisch‐Partenkirchen Medical Center Garmisch‐Partenkirchen Germany

**Keywords:** COVID‐19, diabetic ketoacidosis, DPV database, SARS‐CoV‐2, type 1 diabetes mellitus, 新冠肺炎, 1型糖尿病, 糖尿病酮症酸中毒, 前瞻性糖尿病随访数据, 严重急性呼吸综合征冠状病毒2型

## Abstract

**Background:**

Data on patients with type 1 diabetes mellitus (T1DM) and severe acute respiratory syndrome coronavirus 2 (SARS‐CoV‐2) infections are sparse. This study aimed to investigate the association between SARS‐CoV‐2 infection and T1DM.

**Methods:**

Data from the Prospective Diabetes Follow‐up (DPV) Registry were analyzed for diabetes patients tested for SARS‐CoV‐2 by polymerase chain reaction (PCR) in Germany, Austria, Switzerland, and Luxembourg during January 2020–June 2021, using Wilcoxon rank‐sum and chi‐square tests for continuous and dichotomous variables, adjusted for multiple testing.

**Results:**

Data analysis of 1855 pediatric T1DM patients revealed no differences between asymptomatic/symptomatic infected and SARS‐CoV‐2 negative/positive patients regarding age, new‐onset diabetes, diabetes duration, and body mass index. Glycated hemoglobin A1c (HbA1c) and diabetic ketoacidosis (DKA) rate were not elevated in SARS‐CoV‐2‐positive vs. ‐negative patients. The COVID‐19 manifestation index was 37.5% in individuals with known T1DM, but 57.1% in individuals with new‐onset diabetes. 68.8% of positively tested patients were managed as outpatients/telemedically. Data analysis of 240 adult T1MD patients revealed no differences between positively and negatively tested patients except lower HbA1c. Of these patients, 83.3% had symptomatic infections; 35.7% of positively tested patients were hospitalized.

**Conclusions:**

Our results indicate low morbidity in SARS‐CoV‐2‐infected pediatric T1DM patients. Most patients with known T1DM and SARS‐CoV‐2 infections could be managed as outpatients. However, SARS‐CoV‐2 infection was usually symptomatic if it coincided with new‐onset diabetes. In adult patients, symptomatic SARS‐CoV‐2 infection and hospitalization were associated with age.

## Graphical abstract

**Highlights**

- Diabetic ketoacidosis rate and HbA1c were not elevated in pediatric patients with type 1 diabetes mellitus (T1DM) and severe acute respiratory syndrome coronavirus 2 (SARS‐CoV‐2) infection.
- Pediatric patients with known T1DM did not show an elevated COVID‐19 manifestation index; only pediatric patients with coincident SARS‐CoV‐2 infection and new‐onset diabetes had an elevated rate of symptomatic infection.
- Adult patients showed an age‐dependent increase in symptomatic SARS‐CoV‐2 infections and hospitalization rate.

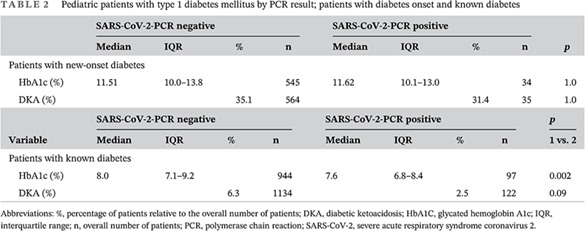

## 摘要

**背景:**

关于1型糖尿病(T1DM)和严重急性呼吸综合征冠状病毒2型(SARS‐CoV‐2)感染患者的数据很少。本研究旨在探讨SARS‐CoV‐2感染与T1DM的关系。

**方法:**

对德国, 奥地利, 瑞士和卢森堡在2020年1月至2021年6月期间进行SARS‐CoV‐2检测的糖尿病患者的前瞻性糖尿病随访(DPV)登记数据进行分析，采用Wilcoxon秩和检验和卡方检验，对连续变量和二分变量进行多重检验。

**结果:**

1,855例儿童T1DM患者的数据分析显示，无症状/有症状感染和SARS‐CoV‐2阴性/阳性患者在年龄, 新发糖尿病, 糖尿病病程和体重指数方面没有差异。SARS‐CoV‐2阳性与阴性患者糖化血红蛋白(HbA1c)和糖尿病酮症酸中毒(DKA)发生率无明显差异。在已知的T1DM患者中，COVID‐19的表现指数为37.5%，而在新发糖尿病患者中为57.1%。68.8%的阳性患者以门诊/远程医疗的形式进行管理。对240例成人T1MD患者的数据分析显示，阳性和阴性患者之间除HbA1c降低外，其他均无差异。83.3%的患者有症状感染，35.7%的阳性患者住院治疗。

**结论:**

SARS‐CoV‐2感染的儿童T1 DM患者的发病率较低。大多数已知的T1糖尿病和SARS‐CoV‐2感染的患者可以作为门诊患者进行管理。然而，如果SARS‐CoV‐2感染与新发糖尿病重合，通常是有症状的。在成人患者中，有症状的SARS‐CoV‐2感染和住院与年龄有关。

## 1 INTRODUCTION

In late 2019, the severe acute respiratory syndrome coronavirus 2 (SARS‐CoV‐2) pandemic began to spread across the world.^1^, ^2^ Growing evidence showed increased morbidity and mortality in patients with type 2 diabetes mellitus.^3^, ^4^, ^5^ On the other hand, data on patients with type 1 diabetes mellitus (T1DM), especially pediatric patients, are sparse and at times inconsistent.^6^, ^7^ In part, conclusions for pediatric patients have been drawn by extrapolation from data on adult patients with diabetes mellitus. This procedure is not valid and may lead to misinformation.^8^ Thus, targeted analysis of pediatric populations as well as adult populations with T1DM is essential in order to gain clarity regarding the impact of SARS‐CoV‐2 infection on these patients. The aim of the present study was to (1) investigate the influence of SARS‐CoV‐2 infections on the course of T1DM and of T1DM on infections with SARS‐CoV‐2 in children and adolescents in Germany, Austria, Switzerland, and Luxembourg, (2) gain information about SARS‐CoV‐2‐infected adult patients with T1DM in central Europe, and (3) learn about the differences between pediatric and adult patients with T1DM.

## 2 METHODS

### 2.1 Data source and study population

Data from the Prospective Diabetes Follow‐up (DPV) Registry were analyzed. The DPV registry is maintained by the Institute of Epidemiology and Medical Biometry at the University of Ulm, Ulm, Germany and collects patient data from diabetes centers in Germany, Austria, Switzerland, and Luxembourg, covering >90% of all pediatric diabetes centers in Germany and Austria.^9^ Informed consent (verbal or written) to participate in the DPV registry was available from patients and/or their parents. The ethics committee of the University of Ulm approved the analysis of the anonymized DPV data (approval no. 314/21).

Data analysis included all T1DM patients aged >6 months with documented polymerase chain reaction (PCR) tests for SARS‐CoV‐2. All patients tested upon inpatient admission as well as outpatients and externally tested patients were included in the analysis. The data collection period was 01/2020–06/2021, and data were analyzed separately for pediatric (age <18 years) and adult (age ≥ 18 years) patients. Data were aggregated per patient ±10 days around the test. Overall, 162 centers contributed to the data collection, including 150 centers from Germany, 10 from Austria, and one each from Switzerland and Luxembourg. Data from SARS‐CoV‐2‐positive and ‐negative T1DM patients were compared. In a second step, the following subgroups of data were further analyzed: First, data from SARS‐CoV‐2‐positive T1DM patients were analyzed, for whom there was documented information on symptoms (comparison of patients with/without symptoms of coronavirus disease 2019 [COVID‐19]). Second, data from hospitalized SARS‐CoV‐2‐positive and ‐negative T1DM patients were compared. Third, a separate analysis of pediatric patients with new‐onset diabetes or follow‐up examinations was performed with respect to glycated hemoglobin A1c (HbA1c) and the rate of diabetic ketoacidosis (DKA). In addition, we analyzed data from SARS‐CoV‐2‐positive and ‐negative adult T1DM patients (total population; hospitalized patients).

### 2.2 Variables

Demographic data included patient age, age at new‐onset diabetes, diabetes duration, sex, and migrant background (patient and/or at least one parent born outside Germany, Austria, Switzerland, or Luxembourg). Clinical data included HbA1c (%, mmol/mol) and body mass index (BMI, kg/m^2^). For the pediatric patients, BMI values were expressed as standard deviation scores (SDS) based on the German KiGGS percentiles (German Health Interview and Examination Survey for Children and Adolescents).^10^ To ensure comparability of the local HbA1c values, the respective values were converted according to the “multiple of the mean” method for normalization to the reference range of the Diabetes Control and Complications Trial (DCCT).^11^ The other clinical parameters were the reason for admission (unknown, new‐onset diabetes, education, DKA, and “other reasons”, e.g., admission for SARS‐CoV‐2 infection), the mode of care (outpatient, inpatient, telemedical care), the proportion of patients with DKA, and DKA at new onset of diabetes. DKA was defined as pH <7.3 and/or bicarbonate levels <15 mmol/L. The COVID‐19 manifestation index was defined as the percentage of symptomatic infections in all patients testing positive for SARS‐CoV‐2.

### 2.3 Statistical analyses

Analyses were performed as unadjusted comparisons using SAS 9.4 software (TS1M7; SAS Institute Inc) on a Windows Server 2019 mainframe to compute the Wilcoxon rank‐sum test for continuous variables and the chi‐square test for dichotomous variables. All analyses were adjusted for multiple testing using the Bonferroni‐Holm method (Bonferroni stepdown). Two‐tailed *p* values <0.05 were considered significant.

## 3 RESULTS

### 3.1 T1DM in children and adolescents

In total, 1855 pediatric T1DM patients underwent SARS‐CoV‐2 testing by PCR; 157 patients tested positive (cumulative positive rate 8.5%). There was no statistical difference between negatively and positively tested patients with respect to age, age at new‐onset diabetes, diabetes duration, and BMI‐SDS (see Table 1). HbA1c was significantly lowered in positively tested patients (*p* = 0.003). The difference was due to the patients with known and treated T1DM; there was no difference in this respect in the patients with new‐onset diabetes. The rate of DKA was not increased in patients who tested positive for SARS‐CoV‐2 compared to patients who tested negative (patients with known diabetes: 2.5% vs. 6.3%, new‐onset diabetes: 31.4% vs. 35.1%; see Table 2).

**TABLE 1:** Pediatric patients with type 1 diabetes mellitus by PCR result

	SARS‐CoV‐2‐PCR negative				SARS‐CoV‐2‐PCR positive				
	Median	IQR	%	n	Median	IQR	%	n	*p*
Age (years)	12.2	8.5–14.9		1698	12.5	8.8–15.3		157	1.00
Age at new onset of diabetes (y)	8.3	4.8–11.4		1698	8.5	5.0–11.1		157	1.00
Duration of diabetes (years)	1.8	0.0–5.5		1698	2.7	0.25–6.0		153	0.27
HbA1c (%)	9.1	7.6–11.1		1489	8.2	6.9–10.1		131	0.003
Body mass index‐SD scores (KiGGS)	0.3	−0.5–1.0		1661	0.4	−0.4–1.1		141	0.66
Male			50.8	1698			47.1	157	1.00
Migrant background (parents/child)			32.7	1698			42.0	157	0.16
Outpatient			24.0	1698			59.2	157	<0.001
Inpatient			75.8	1698			31.2	157	<0.001
Telemedical support			0.2	1698			9.6	157	<0.001
Patients with DKA			15.8	1698			8.9	157	0.17
Patients with DKA at new onset of diabetes			36.1	701			32.1	53	1.00
Inpatients – admission diagnosis									
New‐onset diabetes			41.5	1287			69.4	49	0.001
Diabetes education			41.2	1287			10.2	49	<0.001
Patients with DKA at new onset of diabetes			35.9	632			31.6	38	1.00

Abbreviations: %, percentage of patients relative to the overall number of patients; DKA, diabetic ketoacidosis; HbA1C, glycated hemoglobin A1c; IQR, interquartile range; KiGGS, German Health Interview and Examination Survey for Children and Adolescents; n, overall number of patients; PCR, polymerase chain reaction; SARS‐CoV‐2, severe acute respiratory syndrome coronavirus 2.

**TABLE 2:** Pediatric patients with type 1 diabetes mellitus by PCR result; patients with diabetes onset and known diabetes

	SARS‐CoV‐2‐PCR negative				SARS‐CoV‐2‐PCR positive				*p*
	Median	IQR	%	n	Median	IQR	%	n	
Patients with new‐onset diabetes									
HbA1c (%)	11.51	10.0–13.8		545	11.62	10.1–13.0		34	1.0
DKA (%)			35.1	564			31.4	35	1.0

Variable	SARS‐CoV‐2‐PCR negative				SARS‐CoV‐2‐PCR positive				*p*
	Median	IQR	%	n	Median	IQR	%	n	1 vs. 2
Patients with known diabetes									
HbA1c (%)	8.0	7.1–9.2		944	7.6	6.8–8.4		97	0.002
DKA (%)			6.3	1134			2.5	122	0.09

Abbreviations: %, percentage of patients relative to the overall number of patients; DKA, diabetic ketoacidosis; HbA1C, glycated hemoglobin A1c; IQR, interquartile range; n, overall number of patients; PCR, polymerase chain reaction; SARS‐CoV‐2, severe acute respiratory syndrome coronavirus 2.

SARS‐CoV‐2‐positive patients received mostly inpatient care during the first 6 months of the pandemic (93.3%) whereas negatively tested patients were only treated in 38% as inpatients. As the pandemic went on, this treatment changed. As for 06/2021, patients who tested negative for SARS‐CoV‐2 received care as inpatients in 75.8% of cases, as outpatients in 24% of cases, and by telemedicine in 0.2% of cases. By contrast, only 31.2% of positively tested patients received care as inpatients, 59.3% as outpatients, and another 9.6% by telemedicine (*p* < 0.001 in each case). For SARS‐CoV‐2‐negative patients, the most common reasons for admission were new‐onset diabetes (41.5%) and admission for diabetes education (41.2%). By contrast, 69.4% of patients who tested positive were admitted to hospital due to new‐onset diabetes and only 10.2% were admitted for education, (*p* < 0.001 in each case).

**FIGURE 1:**
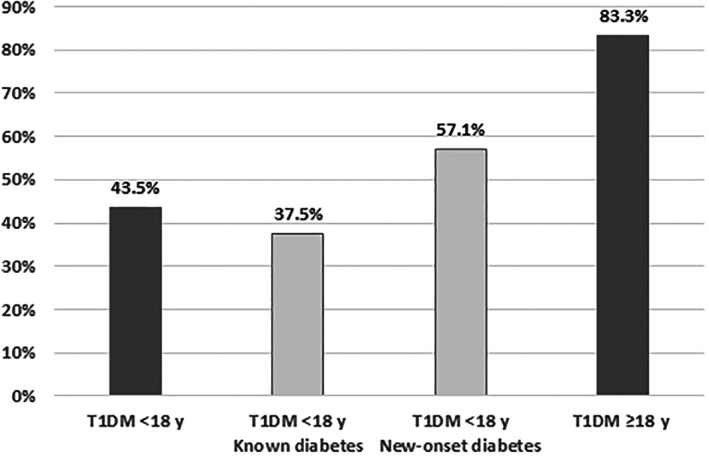
COVID‐19 manifestation index. T1DM, type 1 diabetes mellitus.

Of 46 patients with a positive SARS‐CoV‐2 PCR result and available data on symptoms of SARS‐CoV‐2 infection, 20 (43.5%) had COVID‐19 symptoms. In individuals with known T1DM, the COVID‐19 manifestation index was 37.5% (12/32). By contrast, 8/14 (57.1%) children and adolescents with new‐onset diabetes and 5/6 (83.3%) hospitalized adult patients with new‐onset diabetes showed symptoms of COVID‐19 (Figure 1). There was no significant difference between patients with COVID‐19 and asymptomatic patients with positive SARS‐CoV‐2 PCR results in terms of age, age at new‐onset diabetes, diabetes duration, HbA1c, and BMI‐SDS (see Table 3). 17/26 (65.4%) asymptomatically and 11/20 (55%) symptomatically infected pediatric patients were managed as outpatients. Only 6 (30%) of the symptomatically infected patients were admitted as inpatients, all due to acute diabetes complications (5 patients with new‐onset diabetes and 1 with DKA) and 4 (15.4%) of the asymptomatically infected patients (1 each with new‐onset diabetes/DKA and 2 for diabetes education). 3 (15%) patients with COVID‐19 and 5 (19.2%) asymptomatically infected patients received telemedical instructions. At new‐onset diabetes, DKA was present in 4/8 (50%) patients with symptomatic infection and 2/6 (33.3%) patients with asymptomatic infection.

**TABLE 3:** Pediatric patients with type 1 diabetes mellitus by PCR result and reported COVID‐19 symptoms

	COVID‐19 symptoms: No				COVID‐19 symptoms: Yes				*p*
	Median	IQR	%	n	Median	IQR	%	n	
Age (years)	12.7	8.9–13.9		26	12.3	9.5–14.4		20	1.0
Age at new onset of diabetes (years)	6.9	4.0–9.2		26	7.8	5.3–13.2		20	1.0
Duration of diabetes (years)	4.3	1.1–6.5		26	2.1	0.3–5.6		20	1.0
HbA1c (%)	7.7	6.9–8.8		19	8.5	6.6–10.4		16	1.0
Body mass index‐SD scores (KiGGS)	0.7	−0.3–1.1		23	0.3	−0.1–0.9		16	1.0
Male			61.5	26			35.0	20	1.0
Migrant background (parents/child)			50	26			40.0	20	1.0
Outpatient			65.4	26			55.0	20	1.0
Inpatient			15.4	26			30.0	20	1.0
Telemedical support			19.2	26			15.0	20	1.0
Patients with DKA			7.7	26			15.0	20	1.0
Patients with DKA at new onset of diabetes			33.3	6			50	8	1.0

Abbreviations: %, percentage of patients relative to the overall number of patients; DKA, diabetic ketoacidosis; HbA1C, glycated hemoglobin A1c; IQR, interquartile range; KiGGS, German Health Interview and Examination Survey for Children and Adolescents; *n*, overall number of patients; PCR, polymerase chain reaction.

### 3.2 T1DM in adults

**FIGURE 2:**
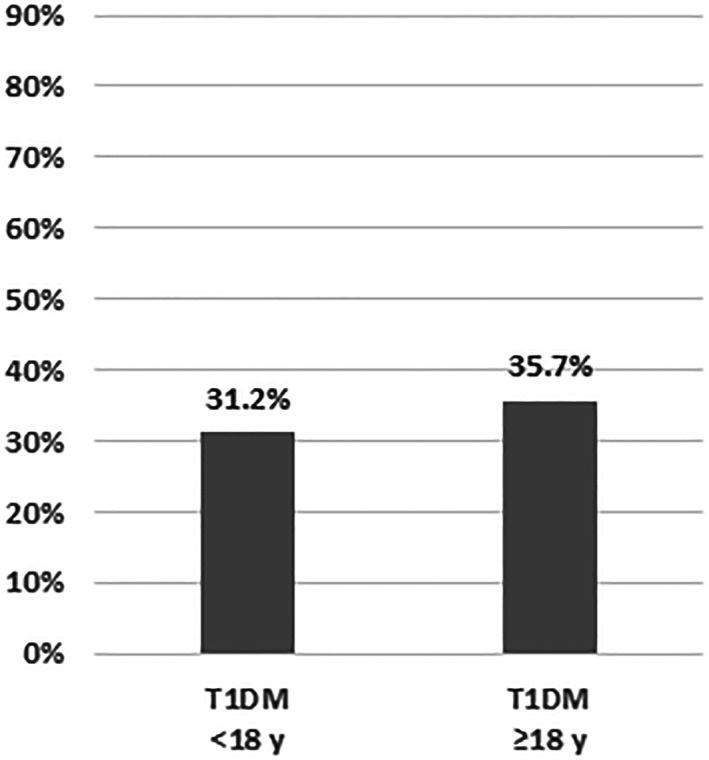
SARS‐CoV‐2 hospitalization rate. T1DM, type 1 diabetes mellitus.

Analysis of the data from 240 adult patients with T1DM showed no statistically significant differences between SARS‐CoV‐2 negatively/positively tested patients except for HbA1c, which was significantly lower in positively tested patients (see Table 4). The most frequent reasons for admission of negatively tested patients to hospital were diabetes education (25.9%), DKA (19.4%), and new‐onset diabetes (14.8%), whereas positively tested patients were mostly admitted for “other reasons” (not primarily diabetes‐related reasons, e.g., SARS‐CoV‐2 infection [35%]) and DKA (30%). Questioning about infection symptoms was documented in 12 patients who tested positive, 10 (83.3%) of whom had symptomatic infection (Figure 1). Nevertheless, only 35.7% of the positively tested patients required hospitalization (Figure 2).

**TABLE 4:** Adult patients with type 1 diabetes mellitus by PCR result

	SARS‐CoV‐2‐PCR negative				SARS‐CoV‐2‐PCR positive				*p*
	Median	IQR	%	n	Median	IQR	%	n	
Age (years)	24.2	19.3–46.4		184	37.1	20.3–55.0		56	0.29
Age at new onset of diabetes (years)	14.3	9.0–27.8		184	15.5	8.4–35.4		56	1.0
Duration of diabetes (years)	10.7	5.8–19.4		184	15.5	9.1–23.1		56	0.31
HbA1c (%)	8.8	7.4–10.3		167	7.6	7.1–8.6		49	0.03
Body mass index	24.3	21.6–27.4		174	24.7	22.3–27.9		48	0.95
Male			54.4	184			64.3	56	0.90
Migrant background (parents/child)			30.4	184			14.3	56	0.20
Outpatient			41.3	184			62.5	56	0.07
Inpatient			58.7	184			35.7	56	0.04
Telemedical support			0.0	184			1.8	56	0.62
Patients with DKA			6.5	184			1.8	56	0.90
Inpatients – admission diagnosis									
New‐onset diabetes			14.8	108			5.0	20	1.0
Diabetes education			25.9	108			20.0	20	1.0
DKA			19.4	108			30.0	20	1.0
Other diagnoses			13.0	108			35	20	0.17
% Patients with DKA			11.1	108			5.0	20	1.0

Abbreviations: %, percentage of patients relative to the overall number of patients; DKA, diabetic ketoacidosis; HbA1C, glycated hemoglobin A1c; IQR, interquartile range; *n*, overall number of patients; PCR, polymerase chain reaction; SARS‐CoV‐2, severe acute respiratory syndrome coronavirus 2.

## 4 DISCUSSION

### 4.1 T1DM in pediatric patients

SARS‐CoV‐2‐positive patients initially received mostly inpatient care during the first 6 months of the pandemic. The data may reflect the initial uncertainty in the management of SARS‐CoV‐2. After initial results suggested that there was no greatly increased risk of disease in young patients with T1DM, care was able to be provided predominantly as outpatient treatment and by telemedicine. In cases in which a pediatric patient with a positive test required inpatient care, this was primarily due to the concurrent new onset of T1DM (69.4%, 34/49 patients).

Our analysis found that SARS‐CoV‐2‐positive patients had significantly lower HbA1c levels than negatively tested patients. Further analysis showed that this difference was due to significantly lower HbA1c levels in patients with known T1DM, while HbA1c levels were the same in positively and negatively SARS‐CoV‐2 tested pediatric patients with new‐onset diabetes. Our database did not contain any information as to why patients were tested for SARS‐CoV‐2, so the reason for the significantly lower HbA1c value in SARS‐CoV‐2 positive patients remains unclear. In the group of SARS‐CoV‐2 negative patients the percentage of patients admitted to the hospital for diabetes education was much higher than in the SARS‐CoV‐2 positive group. The main reason for these admissions was poor diabetes control. This may explain the higher HbA1c level in children and adolescents with negative SARS‐CoV‐2 tests. Population‐wide screening studies among diabetic pediatric patients may provide clarity in this regard. Importantly, no increased HbA1c levels in terms of a risk factor for SARS‐CoV‐2 infection were observed in SARS‐CoV‐2‐positive patients. There were no other significant differences between negatively and positively tested pediatric patients. The results are indicative of the low morbidity in children and adolescents with T1DM and SARS‐CoV‐2 infection, as has also been reported for children from the United States and England.^8^, ^12^ In addition, the data show that metabolic control generally remained stable in the setting of infection and that diabetes‐associated morbidity did not worsen with SARS‐CoV‐2 infection. Our results indicate the success of patient education and outpatient management of patients with T1DM in Germany, Austria, Switzerland, and Luxembourg.

The rate of DKA at new onset of diabetes was significantly higher during the COVID‐19 pandemic than in prior years. Kamrath et al. reported a significantly increased value of 44.7% for 2020.^13^ During the 01/2020–06/2021 analysis period, the DKA rate in our pediatric cohort was still higher than in previous years (35.8% compared to approximately 24%). Possible reasons may include delayed presentation due to fear of visiting the hospital, delayed presentation due to infection with SARS‐CoV‐2, and delayed diagnosis of new‐onset diabetes due to infection symptoms. In their analysis of adult patient data, Pasquel et al. found evidence that infection with SARS‐CoV‐2 itself led to more severe DKA.^14^ Li et al. demonstrated that SARS‐CoV‐2 infection can lead to ketosis and ketoacidosis, not only in patients with diabetes.^15^ In our study population, there was no evidence in this regard, as the rate of DKA was not increased in SARS‐CoV‐2‐positive compared with SARS‐CoV‐2‐negative pediatric patients. Further analysis showed that neither patients with known diabetes nor patients with new‐onset diabetes had an elevated DKA rate compared to patients from the SARS‐CoV‐2‐negative control group.

Among the subgroup of patients with new‐onset diabetes and available data on COVID‐19 symptoms, infection was symptomatic in 8/14 cases (57.1%), which was markedly higher than in the overall study population, where the percentage was 43.5%. This finding indicates that new‐onset diabetes may have aggravated COVID‐19 infection, which is consistent with identical observations by Singh et al.^16^ By contrast, no such evidence was found for patients with previously known T1DM, where the COVID‐19 manifestation index of 37.5% was even below the value of about 50% reported for the general population.^17^

### 4.2 Adult patients

In adult patients with T1DM, the proportion of symptomatic infections was almost twice as high as in pediatric patients (83.3% vs. 43.5%). Definitive statements are not yet available regarding the COVID‐19 manifestation index, which depends on multiple co‐variables, including age, preexisting disease, and socioeconomic status of the population.^18^, ^19^, ^20^ COVID‐19 manifestation index values reported in the literature vary from 55% to >90%^21^, ^22^ and are reported as being about 50% in children,^17^ which is consistent with our data. Age and diabetes duration in SARS‐CoV‐2‐positive adult patients with T1DM tended to be higher than in those with T1DM who tested negative for SARS‐CoV‐2 in our study. This was particularly true for hospitalized patients with SARS‐CoV‐2 infection (24.2 vs. 37.1 vs. 49.2 years). No such associations were observed in our pediatric patients. The hospitalization rate was slightly higher in adult patients with T1DM, even though there were significantly fewer patients with diabetes manifestation in the latter group from DPV network. A separate analysis of the DPV data from adult patients with T2DM, which is not part of the present study, showed an even higher hospitalization rate of 82.3% (median age 73.5 years; unpublished data). This could mean that the sequelae of long‐standing diabetes are risk factors for a symptomatic and/or severe course of SARS‐CoV‐2 infections in adult patients. In addition to the higher age, which in itself is a risk factor for severe SARS‐CoV‐2 infections, this may explain the higher hospitalization rate in adult patients. Indeed, the literature has demonstrated the association between increasing age, cardiovascular risk factors, diabetes sequelae, and a more severe course of the SARS‐CoV‐2 infection.^14^, ^23^, ^24^ From these observations follows the recommendation to lower blood glucose and HbA1c levels as a primary prevention measure to reduce the risk of severe COVID‐19 courses.^25^, ^26^, ^27^ Due to lack of data on COVID‐19 symptoms in adult T1DM patients we were unable to prove this hypothesis with our current data. The significantly higher HbA1c in SARS‐CoV‐2 negatively tested patients compared to positively tested adult patients with T1DM is most likely due to a higher percentage of inpatients with diabetes manifestation (14.8 vs. 5.0%) and diabetes education (25.9 vs. 20.0%).

### 4.3 Limitations

Due to the retrospective design of the study as an analysis of existing database records and the absence of a nondiabetic comparison group, our observations need to be considered tentative and interpreted with caution. Since our analysis compared a relatively small group of patients with SARS‐CoV2‐positive T1DM with a markedly larger group of negatively tested patients, it was repeatedly impossible to measure significant differences between the groups, despite clear differences between the data. Further studies in larger cohorts may be useful to confirm our observations.

Our database did not contain any clear information on the reasons why patients were tested for SARS‐CoV‐2. Since SARS‐CoV2 testing was usually performed on hospital admission but not in the case of outpatient appointments, our data originated mainly from inpatients as well as patients with infection symptoms. This limits the statements regarding the above‐mentioned group. Population‐wide screening studies among diabetic pediatric patients may provide clarity in this regard.

Our results reflect the COVID‐19 pandemic during the periods when the original and the “delta” variants of SARS‐CoV‐2 were predominant. Infection rates were much higher during the subsequent “omicron” wave, especially in pediatric patients, and some symptoms differed considerably. Hence, results for this later period may differ from the present findings. DPV registry data for this period will become available in the fall of 2022.

## 5 CONCLUSIONS

Our results are indicative of the low morbidity in children and adolescents with T1DM and SARS‐CoV‐2 infection. Importantly, our study found no increase in DKA rate among patients with positive SARS‐CoV‐2 tests. Patients who tested positive for SARS‐CoV‐2 did not have elevated HbA1c levels compared to those who tested negative. In addition, no association was found between age/diabetes duration/HbA1c/BMI and symptoms of infections. Thus, no evidence emerged that these factors led to increased SARS‐CoV‐2 morbidity in pediatric patients with a previous diagnosis of T1DM in our study population. Most patients with known T1DM and SARS‐CoV‐2 infection could be managed as outpatients. However, when SARS‐CoV‐2 infection coincided with new‐onset diabetes, the infection was usually symptomatic.

In adult patients, an association was observed between age and frequency of symptomatic SARS‐CoV‐2 infection as well as hospitalization. Since diabetes sequelae are associated with a more severe course, optimization of patients' metabolic situation is recommended.

## AUTHOR CONTRIBUTIONS

B.R.B. was responsible for conceptualization, data analysis and interpretation, investigation, and writing of the original draft of the manuscript. S.R.T. was responsible for methodology, data analysis, formal analysis, and review and editing of the manuscript. C.K. was responsible for review and editing of the manuscript. T.R.R. was responsible for conceptualization, investigation, review and editing of the manuscript. R.W.H. was responsible for conceptualization, supervision, funding acquisition, review and editing of the manuscript. B.K., K.K., A.M., E.M.‐R., F.R., T.R.R. and R.W.H were responsible for data acquisition. S.R.T., C.K., B.K., K.K., E.M.‐R., F.R., T.R.R. and R.W.H. were responsible for scientific discussion of the results and important intellectual content and review and editing of the manuscript. All authors reviewed the draft manuscript for important intellectual content and approved the manuscript prior to submission. S.R.T. and R.W.H. are the guarantors of this work and, as such, had full access to all of the data in the study and take responsibility for the integrity of the data and the accuracy of the data analysis.

## FUNDING INFORMATION

The DPV is supported through the German Federal Ministry for Education and Research within the German Centre for Diabetes Research (grant 82DZD14A02). The funding organization had no role in design and conduct of the study, collection, management, analysis, and interpretation of the data, preparation, review, or approval of the manuscript, or decision to submit the manuscript for publication.

## CONFLICT OF INTEREST

None declared.

## ETHICS STATEMENT

The ethics committee of the University of Ulm approved the analysis of the anonymized DPV data (approval no. 314/21).

## PATIENT CONSENT STATEMENT

Informed consent (verbal or written) to participate in the DPV registry was available from patients and/or their parents.

## Supporting information

Supporting Information.

## Data Availability

The raw data that support the findings of this study are available from the senior author upon reasonable request.
