# Supplementary material for: Type 1 diabetes mellitus and SARS‐CoV‐2 in pediatric and adult patients – Data from the DPV network
Source: J Diabetes. 2022 Nov 28;14(11):758–66. doi: 10.1111/1753-0407.13332 (PMC9705805; doi:10.1111/1753-0407.13332)
Supplement: Supplementary file 1 — Supporting Information. [file JDB-14-758-s001.docx]

**Supplementary information: List of participating DPV centers**

Aachen – Uni-Kinderklinik RWTH, Ahlen St. Franziskus Kinderklinik, Altötting Kinderklinik Zentrum Inn-Salzach, Amberg Kinderklinik St. Marien, Arnsberg-Hüsten Karolinenhospital, Kinderabteilung, Aue Helios Kinderklink, Augsburg Josefinum Kinderklinik, Augsburg Uni-Kinderklinik, Aurich Kinderklinik, Bad Hersfeld Kinderklinik, Bad Kreuznach Diakonie Kinderklinik, Bad Oeynhausen Herz- und Diabeteszentrum NRW, Bad Mergentheim Diabetesklinik, Bautzen Oberlausitz KK, Berlin Charité, Berlin DRK-Kliniken Pädiatrie, Berlin Lichtenberg – Kinderklinik, Bielefeld Kinderarztpraxis, Bielefeld Kinderklinik Gilead, Bocholt Kinderklinik, Bochum Universitätskinderklinik St. Josef, Bonn Uni-Kinderklinik, Braunschweig Kinderarztpraxis, Bremen – Kinderklinik Nord, Bremen Zentralkrankenhaus Kinderklinik, Bremerhaven Kinderklinik, Böblingen Kinderklinik, Celle Kinderklinik, Chemnitz Kinderklinik, Coburg Kinderklinik, Coesfeld Kinderklinik, Darmstadt Kinderklinik Prinz. Margaret, Datteln Vestische Kinderklinik, Deggendorf, Deggendorf Medizinische Klinik II, Dessau Kinderklinik, Detmold Kinderklinik, Dortmund Kinderklinik, Dresden Uni-Kinderklinik, Duisburg Sana Kinderklinik, Duisburg-St. Johannes Helios, Düren-Birkesdorf Kinderklinik, Düsseldorf Uni-Kinderklinik, Erfurt Kinderklinik, Erlangen Uni-Kinderklinik, Essen Elisabeth Kinderklinik, Essen Kinderarztpraxis, Esslingen Klinik für Kinder und Jugendliche, Filderstadt Kinderklinik, Frankfurt Diabeteszentrum Rhein-Main-pädiatrische Diabetologie (Clementine-Hospital), Frankfurt Uni-Kinderklinik, Freiburg Uni-Kinderklinik, Freudenstadt Kinderklinik, Fulda Kinderklinik, Fürth Kinderklinik, Garmisch-Partenkirchen Kinderklinik, Gelnhausen Kinderklinik, Gelsenkirchen Kinderklinik Marienhospital, Gera Kinderklinik, Gießen Uni-Kinderklinik, Greifswald Uni-Kinderklinik, Gummersbach Oberbergklinikum, Görlitz Städtische Kinderklinik, Göttingen Uni-Kinderklinik, Hagen Kinderklinik, Halle Uni-Kinderklinik, Hamburg Altonaer Kinderklinik, Hamburg Kinderklinik Wilhelmstift, Hamburg-Nord Kinder-MVZ, Hameln Kinderklinik, Hamm Kinderklinik, Hanau Kinderklinik, Hannover Kinderklinik MHH, Hannover Kinderklinik auf der Bult, Haren Kinderarztpraxis, Heide Kinderklinik, Heidelberg Uni-Kinderklinik, Heidenheim Kinderklinik, Heilbronn Kinderklinik, Herdecke Kinderklinik, Herford Kinderarztpraxis, Herford Klinikum Kinder & Jugendliche, Hildesheim Bernward Krankenhaus Kinderheilkunde, Hildesheim Kinderklinik, Hildesheim Kinderklinik, Homburg Uni-Kinderklinik Saarland, Itzehoe Kinderklinik, Jena Uni-Kinderklinik, Kaiserslautern Kinderarztpraxis, Kaiserslautern-Westpfalzklinikum Kinderklinik, Karlsburg Klinik für Diabetes & Stoffwechsel, Karlsruhe Städtische Kinderklinik, Kassel Klinikum Kinder- und Jugendmedizin, Kiel Städtische Kinderklinik, Kiel Universitäts-Kinderklinik, Kinderklinik San, Koblenz Kinderklinik Kemperhof, Konstanz Kinderklinik, Krefeld Kinderklinik, Köln Kinderklinik Amsterdamer Straße, Köln Uni-Kinderklinik, Landshut Kinderklink, Leer Klinikum – Klinik Kinder & Jugendmedizin, Leipzig Uni-Kinderklinik, Leverkusen Kinderklinik, Lingen Kinderklinik St. Bonifatius, Lippstadt Evangelische Kinderklinik, Ludwigsburg Kinderklinik, Ludwigshafen Kinderklinik St. Anna-Stift, Lübeck Uni-Kinderklinik, Lüdenscheid Märkische Kliniken – Kinder & Jugendmedizin, Magdeburg Uni-Kinderklinik, Mainz Uni-Kinderklinik, Mannheim Uni-Kinderklinik, Marburg Uni-Kinderklinik, Mechernich Kinderklinik, Meissen Kinderklinik Elblandklinikum, Memmingen Kinderklinik, Minden Kinderklinik, Moers Kinderklinik, München 3. Orden Kinderklinik, München von Haunersche Kinderklinik, München-Gauting Kinderarztzentrum, München-Schwabing Kinderklinik, Münster St. Franziskus Kinderklinik, Münster Uni-Kinderklinik, Neubrandenburg, Neuburg Kinderklinik, Neunkirchen Gemeinschaftspraxis Kinderheilkunde, Neunkirchen Marienhausklinik Kohlhof Kinderklinik, Neuruppin Kinderklinik, Neuss Lukas-Krankenhaus Kinderklinik, Neuwied Kinderklinik Elisabeth, Nürnberg Cnopfsche Kinderklinik, Nürnberg Uniklinik Zentrum f Neugeborene/Kinder & Jugendliche, Oberhausen Kinderklinik, Offenburg Kinderklinik, Oldenburg Kinderklinik, Oldenburg Schwerpunktpraxis Pädiatrie, Osnabrück Christliches Kinderhospital, Paderborn St. Vincenz Kinderklinik, Paderborn St. Vincenz Kinderklinik, Passau Kinderklinik, Pforzheim Kinderklinik, Plauen Vogtlandklinikum, Ravensburg Kinderklink St. Nikolaus, Regensburg Kinderklinik St. Hedwig, Rendsburg Kinderklinik, Reutlingen Kinderklinik, Rheine Mathiasspital Kinderklinik, Rosenheim Kinderklinik, Rostock Uni-Kinderklinik, Rotenburg/Wümme Agaplesion Diakonieklinikum Kinderabteilung, Rüsselsheim Kinderklinik, Rüsselsheim MVZ, Saarbrücken Kinderklinik Winterberg, Schleswig Heliosklinik Kinderklinik, Schweinfurt Kinderklinik, Schwerin Kinderklinik, Schwäbisch Hall Diakonie Kinderklinik, Siegen Kinderklinik, Singen – Hegauklinikum Kinderklinik, Solingen Kinderklinik, Speyer Diakonissen Stiftungskrankenhaus Pädiatrie, St. Augustin Kinderklinik, Stade Kinderklinik, Stolberg Kinderklinik, Stuttgart Olgahospital Kinderklinik, Suhl Kinderklinik, Traunstein Kinderklinik, Trier Kinderklinik der Borromäerinnen, Tübingen Uni-Kinderklinik, Ulm Uni-Kinderklinik, Vechta Kinderklinik, Viersen Kinderkrankenhaus St. Nikolaus, Villingen-Schwenningen Schwarzwald Baar Klinikum Kinderklinik, Waren-Müritz Kinderklinik, Weiden Kinderklinik, Wesel Marienhospital Kinderklinik, Wiesbaden Helios Horst-Schmidt-Kinderkliniken, Wilhelmshaven Klinikum Kinderklinik, Winnenden Rems-Murr Kinderklinik, Witten Kinderarztpraxis, Wittenberg Kinderklinik, Worms Kinderklinik, Wuppertal Kinderklinik.
